# Using social prescribing by Iranian medical students: a step towards community-orientation and social determinants of health by revising the health internship curriculum

**DOI:** 10.1186/s12909-022-03718-8

**Published:** 2022-09-05

**Authors:** Maryam Mazaheri, Maryam Khorramizadeh, Majid Rezai-Rad, Meysam Mard-Soltani

**Affiliations:** 1 Department of Social Medicine and Family, School of Medicine, Dezful University of Medical Sciences, Dezful, Iran; 2 Department of Medical Physics, School of Medicine, Dezful University of Medical Sciences, Dezful, Iran; 3 Department of Healthcare Service Administration, School of Management, Islamic Azad University, South Tehran Branch, Tehran, Iran; 4 Department of Clinical Biochemistry, School of Medicine, Dezful University of Medical Sciences, Dezful, Iran

**Keywords:** Social prescribing (SP), Social determinants of health (SDH), Medical students, Medical education, Curriculum planning

## Abstract

**Background:**

In order to meet patients' social needs, including social prescribing in the curriculum of medical students is a necessity.

**Aim:**

Becoming familiarized with the SDH perspective and referral method to link workers (LWs) and the principles of social prescribing (SP).

**Methods:**

Using Levin's model, the intervention was performed in the field of health of medical students in 2018–2020 for 38 interns in Dezful University of Medical Sciences. Following holding meetings (Plan), a social case was selected and the social version (Act) was developed. Then the other students observed the prescription and identified the strengths and weaknesses (Reflect). Finally, the results were evaluated by Kirk Patrick model.

**Results:**

At the reaction level, 63.14% agreed with the applicability of SP and 68.42% with its usefulness for prospective work; 97.36% believed that familiarity with the community context was essential and 78.93% considered SDH study and SP’s necessary. At the learning level, over 90% of the total score was obtained. The results of behavior level included interest, compassion and following up people's problems, the level of results, empowerment and improving unhealthy living conditions of individuals.

**Conclusion:**

Promising positive results indicate that SP can be one of the ways of supporting primary health care.

## Introduction

Recent estimates attribute 10 to 20 percent of health outcomes to medical care, 30 percent to genetics, 40 to 50 percent to behavior, and 20 percent to the social and physical environment. Individual behavior and the environment are often studied together as the non-medical determinants of health. In studies that only consider modifiable determinants and ignore genetics, the non-medical factors account for 80 to 90 percent of a person’s health, and the contribution of medical care remains 10 to 20 percent [1].

An approach that targets the social, economic, and psychological risk factors that lead to preventable diseases is social prescribing [2]. One such intervention is social prescribing, which helps patients to access non-clinical sources of support primarily, but not exclusively, within the community sector [3]. Social prescribing is the modification of unhealthy lifestyles and conditions using social institutions and charities. Prescribing this kind of prescription reduces the number of patients and by receiving non-drug interventions, the amount of their drugs is reduced and eventually their condition improves. In SP, patients with unhealthy living conditions are introduced to donors, volunteer citizens, charities and grassroots organizations related to social health determinants (Alderwick et al. 2018). Dr. Pezeshki, in 2019, proposed social prescribing (SP) as a cost reduction policy to the Ministry of Health, and stated that many mental and physical patients who suffer some disorders in their living conditions, and partial or thorough improvement of these disorders may lead to thorough treatment of patients or at least slow down the trend of the disease progress and improve the patients' prognosis. SP means modifying unhealthy lifestyles and conditions using social institutions and charities while reducing the number of patients through taking benefit from non-pharmacological interventions [4]. Some studies indicate that SP significantly reduces the burden of patients' visits to clinics and hospitals, and consequently the costs of diagnosis and treatment [5, 6].

In Iran, in the fourth phase of the health system transformation plan (2014), transformation and innovation packages in medical education were developed and communicated. In these packages, responsive and justice-oriented education, was emphasized to meet the needs of society [7]. The contents of medical education programs are generally disease-oriented and healthcare has been neglected [7]. A lot of efforts have been made in western countries to revise the curriculum of medical students in line with changes and demographic needs, as well as technology. SP has been added to the curriculum of medical students in the universities of England, America, Scotland and Ireland [8].

Dezful where the research was done is an agricultural area. There are a few administrative and government centers in the suburbs and surrounding villages. Therefore, people are engaged in agriculture, animal husbandry and other self-employed jobs. Culturally, most of the people of Dezful villages due to the great distance from the city and its dispersion, have special and regional subcultures. In terms of of social determinants of health and disease, they are exposed to different diseases. Their access to healthcare services related to these problems such as counseling centers, entrepreneur centers, professional technical centers, food providers in different nutrition groups, social support groups, etc. are in great distress. And sometimes they can afford to travel to the City center to receive these services. Most of the time, due to these social problems, the incidence of psychosomatic diseases increases and it is necessary for the local physicians to be aware of social problems so that he/she does not prescribe additional medical prescriptions and cause more harm to patients due to excessive and unnecessary medication and paraclinical services. Even for people living in the city center, this can often be a problem due to a lack of knowledge of the places and organizations mentioned above, or even not being able to recognize their problems. Therefore, physicians need to be aware of this issue, refer patients to those who can help recognize their problems, and prevent patients from physically, mentally, and financially getting harmed. We noticed that medical students and even physicians in the area were not familiar with the social problems of families referring to them.

**Fig. 1:**
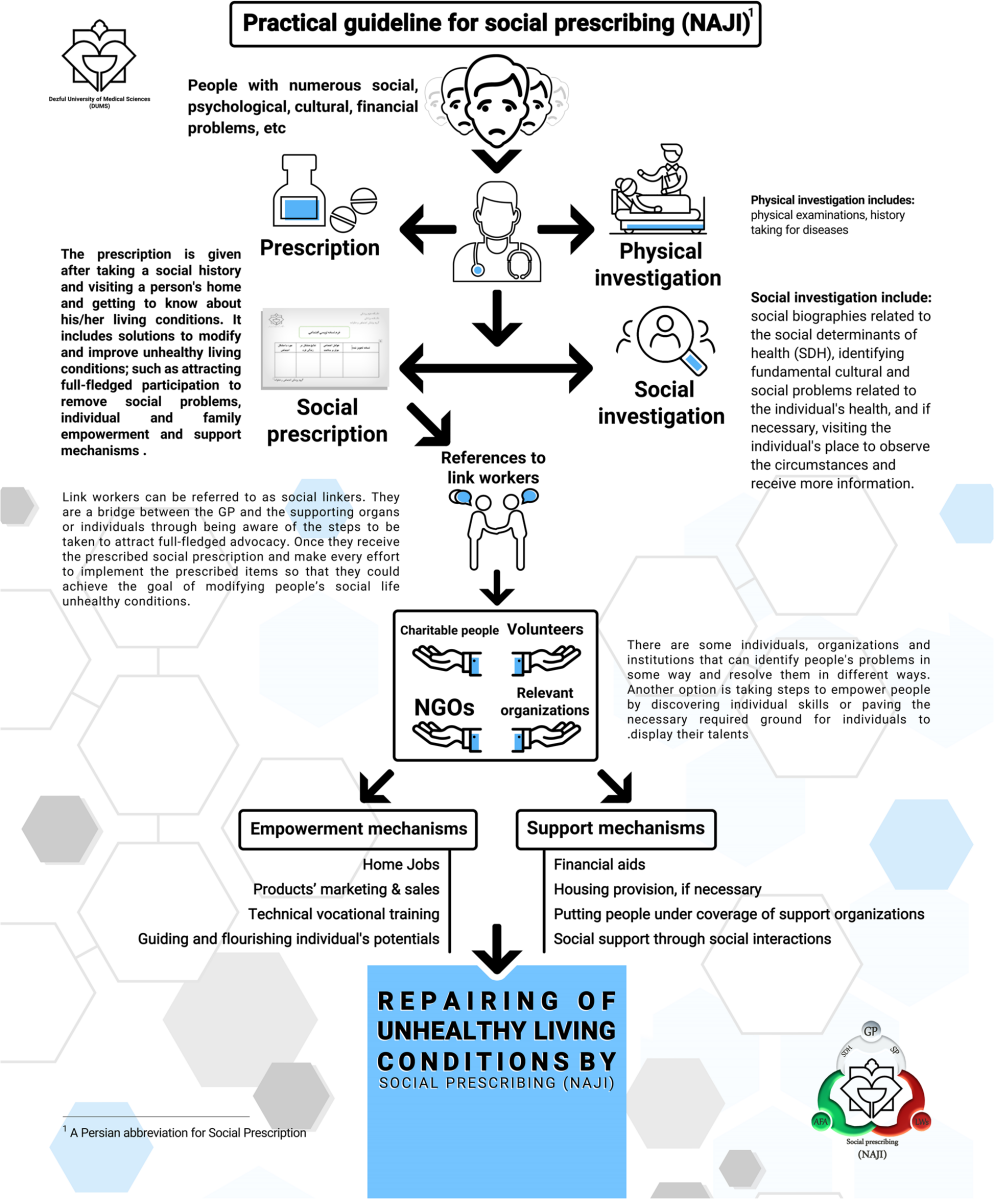
Practical Guideline for Social Prescribing (SP) designed by Dezful University of Medical Sciences (DUMS)

Whereas general practitioners (GPs) are at the frontline of healthcare, the need to include SP in the curriculum of medical students in order to socialize health was severely felt. Therefore, the present study was conducted to familiarize and train practitioners with a sociological vision in order to understand the social context of patients, and non-pharmaceutical therapies and the principles of SP. The philosophy behind it was making practitioners familiar with category so that they could understand people's social problems and prevent them from too much referring to clinics and medical centers, due to clinical manifestations emanated from mental, psychological and social problems. This may provide medical services to people who are really in need of them, and solve people's problems radically and reduce the use of their extra medications. More information about pratical guidelines for SP is shown in Fig. 1.

## Methods

This study was conducted in the medical school of Dezful University of Medical Sciences (DUMS) for all students of general medicine attending internship during the years of 2018- 2020 for three semesters. It was aimed at designing and implementing a development plan, knowledge-enhancing, skills training and empowerment program for prospective practitioners. According to the curriculum, medical internship students will attend comprehensive health service centers for one month to fulfil their line health, as scheduled. This study was conducted during this course.

### Action research approach

**Fig. 2:**
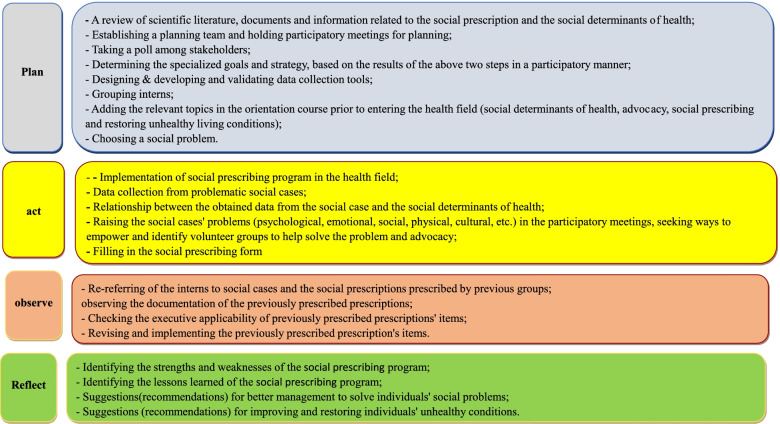
Measures taken in accordance with action research model (planning, acting, observing and reflecting)

This approach was used to implement the SP program and empower students to recognize SDH. The task was performed in four steps: planning, acting, observing and reflecting [9]. In addition, measures taken in this study in accordance with this model are shown in Fig. 2.

### Evaluation strategy

#### Kirk Patrick evaluation model

**Fig. 3:**
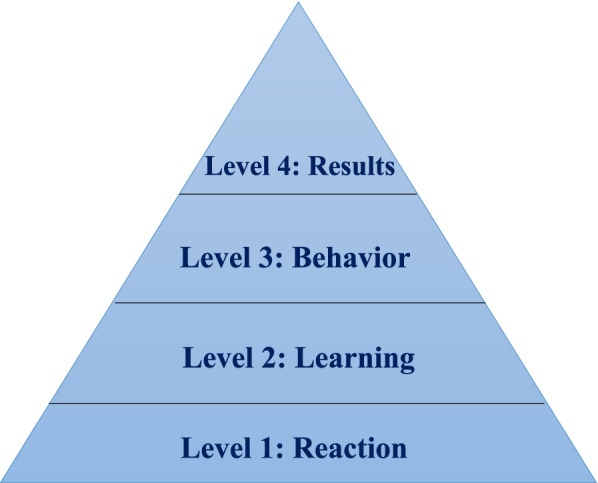
A general view of Kirk Patrick evaluation model (Adapted from: [10])

In order to evaluate the program four levels of Kirk Patrick model (reaction, learning, behavior and results) were used (Fig. 3).

##### Reaction level

At this level, a researcher-made questionnaire was designed with 5 multiple-choice questions on the Likert scale and 5 descriptive questions. After making the revisions in recommended validity test and testing the reliability of the questionnaire, at the end of the course students were asked to fill in the questionnaire anonymously.

##### Level of learning

At this level, to assess the learning rate of the participants [10], the information obtained from students during the internship was used. For this purpose, the obtained data were presented in several formal sessions called "case presentation sessions" while faculty officials, group members and health center officials were in attendance. In these meetings, once the representatives of each group handed over the portfolio, which included all the program documents, presented the measures taken and read the text of SP in the presence of other groups of interns. At the end, the faculty members presented their views and the students answered the questions and their score, 40% of the total score of the course, was calculated.

##### Level of behavior

To determine the changes in behavior, field observations, field notes, student reports and their feedback were used.

##### Level of results

In order to determine the results, the impacts of the prescribed SPs for the students and their actions in the form of notes in the field and students' written and oral reports were used.

### Data analysis

Quantitative data obtained from the checklist were analysed using descriptive statistics and central and dispersion indices (mean and SD) through SPSS 16. For the data obtained from the qualitative section and to reach the study themes, three experts studied the topics and the students' answers, and made sure that the extracted data were correct.

## Results

After implementation of the program, two qualitative and quantitative dimensions were obtained from the participants' experience. The program designing was done in the first step of action research (the planning step), and even prior to that. In this step, with the assistance and opinions of the team members, the necessary arrangements were made and a general plan with specific topics in accordance with the curriculum was designed to be implemented. In addition to the required explanations presented in the orientation meeting, as well as monitoring meetings on the issue utilizing applied examples during interviews with selected cases and other data collection methods, it also led to the enhancement knowledge level of internships of medical students on social factors impacting health and its relevant effects. And by asking about the manner of collecting data and communicating with cases, as well as asking about manner of developing SP, we were ensured that the level of their knowledge was upgraded.

Implementing the program in the village structure enabled medical students to get fully acquainted with the ‘village life structure’. The whole educational study was conducted in the population under the auspices of two Comprehensive Health Service Centers linked with DUMS. One of the consequences of running this program was manner of attracting full-scale participation. To this end, in order to achieve goals of the SP students had to attract general participation.

In order to enhance the level of knowledge and skills of the medical students during the internship on the principles and method of developing SP, the necessary materials were explained in detail in the briefing meeting. After ensuring that the theoretical content was learned, the SP program and the process were done step by step in the field, monitored and the students' questions were answered, and the understanding problems on how to write the SP were solved. The copies were written in full.

Evaluation of the SP curriculum with emphasis on social factors affecting health in the internship health curriculum was done through using Kirk Patrick model. The results obtained in each step are as follows:

### Reaction level

A survey form was designed for the first level. In this study, given the applicability of the SP scheme, the views of 38 medical students on the effectiveness of the plan, strengths and weaknesses and suggestions are shown in Table 1.

**Table 1:** Relative frequency of students' answers on the health field internship on the social prescribing (SP) program

Dimensions of effectiveness of the Scheme	Strongly agree (%)	Agree (%)	No Idea (%)	Disagree (%)	Strongly disagree (%)
In my view SP was quite operational	15.78	47.36	10.52	10.52	15.78
What I learnt in the program is applicable in my prospective career	18.42	50.0	13.15	7.89	10.52
In my view, dealing with the issue in the internship program is essential	10.52	42.1	7.89	26.31	13.15
In my view, GPs need to know their community, their patients and LWs very well	60.52	36.84	2.63	0	0
In my view, cognizance of social determinants effective in health are essential for SP	31.57	47.36	18.42	0	2.63

In order to receive students' feedback with regard to implementing the social prescription task, open-ended questions were raised that examined and evaluated the learnings, strengths and weaknesses of the program, its application, and suggestions. Examining the descriptive responses of this part indicated that students have learned new things; for instance, there is no deeper knowledge and understanding of people's social issues and problems, social determinants of health, people’s mental and physical problems, the importance of link workers (who do not exist in Iran and could be very helpful if such a position is created) and referrals of people with problems to benefactors and NGOs and volunteer groups. The students were satisfied with reaching understanding in these cases and believed that it could be effective in their medical profession.

### Level of learning

In this step, case presentation meetings were held and groups of students presented the measures taken by themselves in several sessions. The score of SP assignment accounted for 40% of the total internship score. The score given to each group at the end of the sessions according to the measures taken and sending the relevant files and documents with the rest of the logbook scores and forms given were accumulated and the training department was provided with their score out of 20 (Table 2).

**Table 2:** Social prescribing assignment scores, total scores and their average

	**Case presentation score out of 40**	**Total score of the year of admittance out of 20**
Average	36.02	18.16

### Level of behavior and level of results

**Fig. 4:**
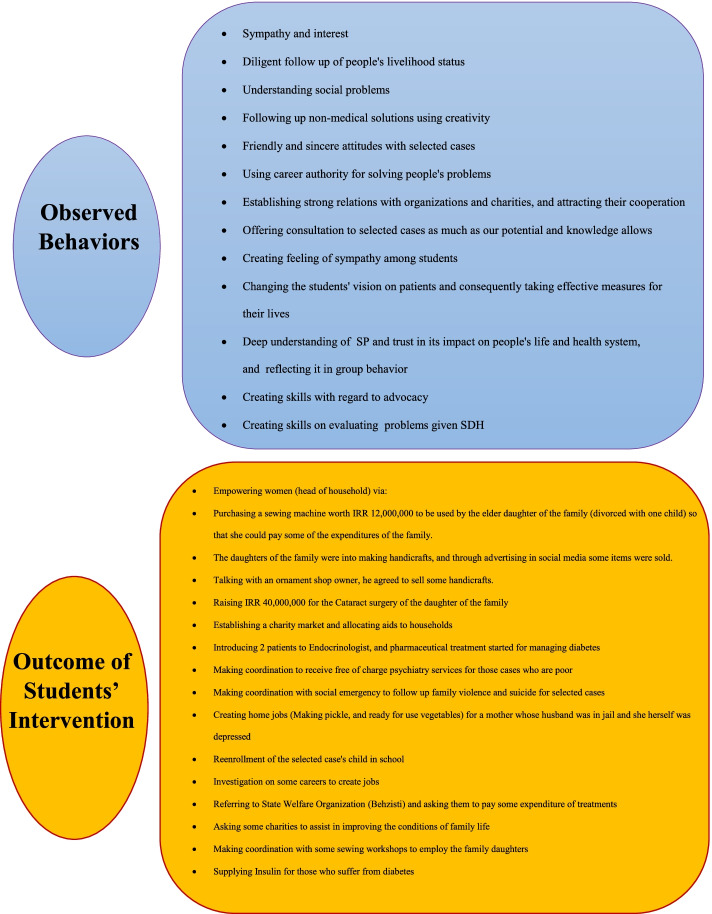
Results achieved from level of observed behaviors and outcomes of students’ intervention after developing social prescribing (SP)

To measure level 3, it was intended that students return to their workplaces after graduation to observe their behavioral changes. However, the idea did not come true due to reasons such as: students not being local and not being available after entering the labor market, not being graduated within the study period and also lack of legal and environmental support for prospective practitioners to perform the desired behavior. Therefore, Fig. 4 refers to the observed behaviors of students during the one-month internship and developing SP program.

It should be noted that in order to support students and strengthen the motivation to develop appropriate behaviors, the Department of Social Medicine of the university made every effort to make the necessary arrangements for work progress, companionship with students, intellectual assistance and elimination of executive shortcomings of students. Finally, due to the recording of higher scores for more active and successful students, the motivation of the next students increased. Whereas this work was in a way related to a class assignment, and students might compare themselves with students in other universities and cooperate less, the following measures were thought of:

1. Accompanying students with regard to training them on the manner of collecting data, interviewing people, viewing documents and providing required recommendations.
2. Intellectual companionship and challenging students' minds to explore avenues to help people in order to write a social prescription.
3. Collaborating with students in implementing versions of social prescription written about some people and encouraging them to implement actions in the best possible way.
4. Allocating more points to useful and executive actions of the written versions of social prescription in order to create more motivation.
5. Enhancing students' motivation level and strengthening their behaviors through noticing people's satisfaction and realizing that the results of the actions taken were satisfactory (implementing social prescription).

Measuring level 4 also requires time and a general review of national indicators with regard to reducing treatment costs and enhancing patients' life quality, which was very difficult and time consuming to measure. However, as groups of students had voluntarily taken plenty of non-pharmaceutical measures and interventions for the selected cases after developing SP, they reported the results (Fig. 4).

## Discussion

Health determinants are new challenges. Issues such as societal security, social relations, food, income, women's empowerment, respecting human rights and equality have been considered. Chief among these factors is poverty, the biggest threat to health. Trend of demographic changes such as urbanization, increasing number of elderly people and high prevalence of chronic diseases have created new problems for all countries. Other social, behavioral, and biological changes, such as lifestyle changes, resistance to antibiotics and other medications have led to increasing drug abuse and domestic violence, and thus threatening the health and well-being of hundreds of millions of people worldwide. New and reemerging infectious diseases, as well as paying more attention to mental illnesses, require immediate action. In order to adapt to changes it is vital to evolve health promotion approaches in health determinants.

The SP program for medical students was designed for the first time in Iran and in the medical school of DUMS during a health internship course. By designing and implementing this program as a team, the students became closely acquainted with the village context and their knowledge of social issues was enhanced. During the implementation of the program, students also learned how to get involved and provide comprehensive support and communication with donors, sponsors and volunteers, and how to improve their skills in this area. Therefore, the idea came true through taking a social history, and identifying people who may take benefit from proper communication, and solve social problems and reshuffle unhealthy living conditions of patients.

The Ottawa Charter recommends that you seek support and mediation to empower people; therefore, health promotion emphasizes achieving justice in health. This goal includes providing supportive environments, access to information, acquiring living skills, and opportunities to create healthy choices. People cannot reach the highest level of health unless they have control over their own SDH [11].

Regarding the impacts of this program in the UK, Hamilton-West et al. showed that goal-based value may contribute to this issue [12], which is consistent with the results of our study.

Chiva Giurca study also concluded that the patient's needs should be identified by physicians, and it requires new tools. SP is a simple and sustainable way to identify social factors impacting health. When conventional medical treatments fail to manage some cases; other methods should be used. This reduces the pressure on services and is a great opportunity for students and prospective physicians to acquire skills. These skills affect the lives of people in the community [8]. These findings are consistent with what medical students referred to in the present study.

The evaluation of the program implemented based on the Kirk Patrick model in 4 levels showed that this program has managed to achieve its predetermined goals. More than half of the medical students agreed with the practicality and usefulness of the SP program (63.14%) and 52.62% stressed the necessity of embedding this program in the curriculum. In the study conducted by Santoni et al., 98% of students considered SP to be feasible and useful for their career in future. They believed that new strategies were needed for this issue and could play a key role in shaping the beliefs of prospective physicians. The majority was eager to get to know more about this, and in their view it should be included and emphasized in their routine curriculum. According to the students participating in this study, interaction with people is a prominent factor and the relevant skills should be taught practically, so as not to be forgotten. They also suggested this program to be frequently implemented in various general practice courses and sections, as well as for educational programs in specialized fields of cancer and the elderly. They also reiterated the long-term support and prominence of primary health care in this program [13].

97.36% of students agreed with the issue of familiarity with the community context and SDH. Perhaps this issue can be justified by the fact that due to the impacts of Iran’s’Education Transformation Plan and the Rural Insurance Plan’ and the perspective of the community-oriented physician and meeting the needs of society, the physicians' perception over recent years has changed, and physicians have addressed this issue. They have realized that cognizance of the context of community may further contribute to their therapeutic role and also reduce their workload. The percentage of opponents of this case and the SP was insubstantial (3.0%). This finding was similar to SDH training and SP in the UK [8].

Therefore, it could be concluded that recognizing SDH and referring chronic patients in need of non-pharmaceutical treatments by physicians to LWs and those who could help them by resolving people's social problems is a priority. Of course, LWs need to have great non-clinical and practical skills, social relationships and high motivation, which requires further research [14]. The absence of LWs in the Iranian healthcare system and its obscurity may be other reasons. Defining this position along with the relevant job description, as well as training courses requires regular and in-depth planning in this regard.

The Ottawa Charter states: ‘Our societies are complex and interrelated. Health cannot be separated from other goals. The inextricable links between people and their environment constitute the basis for a socioecological approach to health.’ Health is a positive concept that emphasizes social and personal resources, as well as physical abilities. Therefore, promoting health is not only the duty of the health sector, but also goes beyond paying attention to a healthy lifestyle and extends to the well-being of people in society. Health prerequisites of basic health conditions and resources include shelter, education, food, income, and a sustainable ecosystem. Improving community health requires investment in these basic prerequisites [11].

The students pointed out the weaknesses of the program, such as: the short duration of the course and the lack of sufficient opportunity to address this issue, the lack of a clear definition of LWs duties, lack of necessary infrastructure, low cooperation of people and organizations and donors in this field and insufficient funding. Given the fact that this program is the first of its kind implemented in a small city and in a fledgling medical science university in Iran, it will undoubtedly have numerous weaknesses. In this regard it is our suggestion to repeat the task in other universities and find its weaknesses in the implementation of this plan, so that through removing the weaknesses and establishing cohesive interested teams, measures could be taken to solve people's non-pharmaceutical problems. Meanwhile, whereas this program is merely aims to make medical students acquainted with work and how to solve problems in the community and to get acquainted with LWs, etc., and practitioners are not responsible for it at all (of course, students were not aware of this issue) and felt they were inexperienced and unprofessional in this area; while this is duty of LWs. After being identified and after passing training courses and once their duties are described, the LWs should rush to assist physicians. In this study, Behvarzes who are fully acquainted with the village context and know people and their living conditions, the donors and related organizations in the village, played the role of LWs.

Other studies have also shown that effectiveness in line with releasing capacity requires significant resources and is not quickly eliminated [15], and to measure public health interventions and the social impact learning process, the value of SP and other aspects of the study need to be determined.

Using powerful tools to study living conditions, welfare systems, and social impacts on health through interviews and observations and focus groups is costly. In order to implement appropriate NHS services by practitioners with the guidance and practice of SP and geographic coverage of required services and to make SP operational, local changes, required level and source of funding, referral sources, scope of activities performed, and goals need to be taken into consideration [5]. This requires strong infrastructure, and generally speaking strong healthcare systems struggle with severe cost and resource problems, and budget and proper infrastructure. However, there are promising findings and valuable actions of prospective practitioners, and other studies stress the impact and determination of the program with bright and positive results on people's lives [6, 14].

Students considered issues such as sense of altruism, familiarity with different cultures and people from various classes of society, NGOs, benefactors and social factors affecting health as useful and strengths of the program.

Results of Levels 3 and 4 of the Kirk Patrick evaluation model indicated that students eagerly followed this program and while deeply understanding people's social problems and believing in the impact of SP on people's lives and healthcare system and pursuing non-pharmaceutical solutions using their creativity and establishing strong relationships with organizations and donors and engaging in all-out participation voluntarily implemented SP. The results of their actions led to valuable measures such as: empowerment of women heads of households, psychological and social support, pursuit of domestic violence and suicide through getting help from social emergencies, creating job opportunities, continuing education and improving the living environment. In similar studies, the results have been also successful. Carnes et al. study revealed that the SP, along with a large number of existing organizations of which we are unaware, reduces the rate of practitioners' consultation. Social self-efficacy also reduces the need to see a doctor, which is a social asset and should be highlighted as an overall national strategy [14].

In Northern Ireland, Loftus et al. reported the impacts of SP with non-pharmaceutical interventions on reducing isolation and the use of secondary medical and emergency care, as well as improving the management of patients with numerous social complexities. Patients continued education and quality of their mood and self-esteem improved, and they finally came to the conclusion that physicians need to further and better understand chronic diseases and their social problems [15].

According to Morton et al., at the moment there the perception is that a non-clinical framework is needed for mental illness. Links and local resources may contribute to solving this problem. SP is helpful for resilience and life-enhancing services, and has lots of benefits for people, particularly for those living in the suburbs. In addition, the NHS-based social prescribing initiative should be developed to ensure availability of justice [16]. Moffatt et al. also suggested that, whereas changes are complex, time-consuming, effective interventions should be made available to policymakers and governments, and that SP should be considered as complementary to healthcare [17].

Studies reveal that given the promising positive results and the potential to meet social needs, SP may be introduced as one of the ways to support PHC. Providing opportunities for interaction and job enrichment leads to health promotion and may be considered a global intervention in reducing the depletion of human and social resources. SP services mean that rather than finding community-oriented solutions, one should think of changing healthcare systems. In order to minimize the workload of practitioners, people's awareness of basic needs should be improved [6].

Of course, in general, it should be kept in mind that job creation programs along with the support of the government and holding recreational, educational and training programs may take a big step towards solving numerous social problems of people with different backgrounds, in case the root problems are solved.

One of the limitations of this study was the lack of a control group, which has been observed in other similar studies as well [6]. However, this is a scholarship of teaching and learning (SOTL) study. It means the aim was to install an educational intervention in our university to enhance the quality of education. From what we know of the literature In educational studies, field studies, using control and experimental groups is difficult. Fortunately, there are alternatives and we can evaluate the outcomes of this kind of interventions using various evaluation methods that are acceptable in the literature of education [18].

## Conclusion

Achieving the highest level of well-being or physical, mental and social health requires individuals or groups to have the ability to identify and fulfill their desires and needs and be able to either change their environment or cope with it. This is true of men and women alike. Mediation to meet the needs and vision of health is not just provided through the health sector. Most importantly, health promotion includes coordinated activities of governments, the health sector and other social and economic sectors, non-governmental and voluntary organizations, local authorities, industry and public media. The Ottawa Charter emphasizes the necessity of developing individual skills. Health promotion supports individual and social development through providing health education information and enhancing living skills. Fulfilling this leads to availability of more options for people to control their health and environment and further improve their health. At the same time, ground should be paved in a way to enable people to acquire knowledge throughout their lives to prepare themselves for all stages of life. The activities of educational institutions, voluntary business professions and institutions are essential in this field [11]. One of the steps to achieve the above-mentioned goal is for prospective physicians to do this with the cooperation of LWs in the light of legal and political support and creating a suitable platform with multiple links. This should be done from the time students are attending university.

## Data Availability

The datasets used and/or analyzed during the current study are available from the corresponding author upon reasonable request.

## Abbreviations

SP: Social prescribing
SDH: Social determinants of health
LWs: Link workers
GP: General practitioner
